# Estimating the infection burden of COVID-19 in Malaysia

**DOI:** 10.1371/journal.pntd.0010887

**Published:** 2022-11-08

**Authors:** Vivek Jason Jayaraj, Chiu-Wan Ng, Awang Bulgiba, Maheshwara Rao Appannan, Sanjay Rampal

**Affiliations:** 1 Centre for Epidemiology and Evidence-based Practice, Department of Social and Preventive Medicine, Faculty of Medicine, University of Malaya, Kuala Lumpur, Malaysia; 2 Ministry of Health Malaysia, Putrajaya, Malaysia; Universidade do Estado do Para: Universidade do Estado do Para, BRAZIL

## Abstract

Malaysia has reported 2.75 million cases and 31,485 deaths as of 30 December 2021. Underestimation remains an issue due to the underdiagnosis of mild and asymptomatic cases. We aimed to estimate the burden of COVID-19 cases in Malaysia based on an adjusted case fatality rate (aCFR). Data on reported cases and mortalities were collated from the Ministry of Health official GitHub between 1 March 2020 and 30 December 2021. We estimated the total and age-stratified monthly incidence rates, mortality rates, and aCFR. Estimated new infections were inferred from the age-stratified aCFR. The total estimated infections between 1 March 2020 and 30 December 2021 was 9,955,000-cases (95% CI: 6,626,000–18,985,000). The proportion of COVID-19 infections in ages 0–11, 12–17, 18–50, 51–65, and above 65 years were 19.9% (n = 1,982,000), 2.4% (n = 236,000), 66.1% (n = 6,577,000), 9.1% (n = 901,000), 2.6% (n = 256,000), respectively. Approximately 32.8% of the total population in Malaysia was estimated to have been infected with COVID-19 by the end of December 2021. These estimations highlight a more accurate infection burden in Malaysia. It provides the first national-level prevalence estimates in Malaysia that adjusted for underdiagnosis. Naturally acquired community immunity has increased, but approximately 68.1% of the population remains susceptible. Population estimates of the infection burden are critical to determine the need for booster doses and calibration of public health measures.

## Author summary

The global scale of COVID-19 is unprecedented. Malaysia has experienced several waves of infection, leading to an overwhelmed health system. Nonetheless, reported cases remain an inaccurate estimate of the true disease burden within the population. We approximated the infection fatality ratio using an estimated adjusted age-specific case fatality ratio. We then estimate the true number of infections that have occurred in Malaysia. A total of 9.95 million cases are estimated to have occurred amongst Malaysians between March 2020 and December 2021. This figure corresponds to 32.8% of the total population of Malaysians. These findings suggest that community immunity remains low, and a great deal of the population remains susceptible. Booster doses and periodic strong public health measures may be required for the foreseeable future.

## Introduction

The global transmission of COVID-19 is unprecedented and has led to more than 282 million cases and 5.4 million deaths as of 31 December 2021 [1]. Efforts to contain its transmission have focused primarily on public health and social measures (PHSM) that have come at significant economic and social costs [2].

Malaysia has reported 2.75 million cases and 31,485 deaths as of 31 December 2021 [3]. The first large outbreak in Malaysia was managed successfully using movement restrictions between March and April 2020 [4]. However, since September 2020, institutional outbreaks, state elections, and inconsistent implementation of PHSM have led to large periodic outbreaks [5].

Underestimation remains an issue despite the substantial reported burden of disease. Screening strategies and diagnostic test accuracy are two factors that drive this underestimation [6]. Reported cases are biased estimators of true disease burden. The true burden of disease may be estimated using seroprevalence surveys and random sampling [6,7]. Alternative indicators such as hospitalization and emergency room data do not estimate the overall infection rate [7]. A more accurate estimator of the actual COVID-19 infection burden may be COVID-19 mortalities, especially in countries with low excess mortalities [7–9].

Accurately estimating the epidemic size is critical in forming situational awareness in designing and evaluating future public health and social measures. Misclassified estimates of total COVID-19 cases may hamper forecasting and future disease control planning [9,10].

To the best of our knowledge, no studies have yet estimated the true burden of disease at a population level in Malaysia. We aimed to estimate the burden of COVID-19 infections in Malaysia between 1 March 2020 and 31 December 2021.

## Methods

### Ethics statement

This study was registered under the National Medical Research Register with a registration ID (NMRR-20-1208-55087) and obtained ethical approval from the Medical Research and Ethics Committee (MREC), Ministry of Health Malaysia.

### Data source and study population

A retrospective cohort study design was utilized. Data on cases and mortalities were extracted from line lists maintained by the Ministry of Health, Malaysia, between 1 March 2020 and 30 November 2021 [11]. Healthcare facilities adjudicate all deaths as either death due to COVID-19 or death with COVID-19 based on a set of criteria (S1 Appendix)- with only the former contributing to COVID-19 mortality statistics. Age-stratified population projection estimates for 2021 were extracted from the Department of Statistics, Malaysia (DOSM) [12]. Additionally, we extracted line lists of age-stratified all-cause mortalities from the DOSM between 1 January 2020 and 30 September 2021. We excluded non-Malaysians from this study due to possibly differential health-seeking behavior and uncertainty in the enumeration of non-Malaysians in Malaysia due to undocumented immigrants [13].

### Statistical analysis

Data were explored for missingness using descriptive statistics, visualizations, and a univariate and multivariate logistic regression model. A multiple imputation model using expectation-maximization with bootstrapping was utilized to impute age. Age was then categorized as 0–11, 12–17, 18–50, 51–65, and >65 years old.

Cases, deaths, brought-in dead, incidence rates, mortality rates, and adjusted case fatality rates (aCFR) estimates were tabulated cumulatively and stratified by age and time. Death location was classified as either in-hospital death or brought-in-dead. A 95% confidence interval around these parameters was estimated using Wald’s bootstrapping approach [14]. The daily incidence and mortality rates were visualized to explore longitudinal trends within Malaysia. The mid-year population was assumed to be the population at risk for the risk set on each day. The incidence density (date of reporting) and mortality rate (date of actual death) are given by:

$$Incidence\;density\;(per\;100,000\;population)=\frac{No.\;of\;reported\;COVID-19\;cases\;(in\;time\;period)}{Mid-year\;population}\;x\;100,000$$

$$Mortality\;rate\;(per\;100,000\;population)=\frac{No.\;of\;reported\;COVID-19\;mortalities\;(in\;time\;period)}{Mid-year\;population}\;x\;100,000$$

The reported case fatality rate (CFR) is estimated as the percentage of COVID-19 mortalities on a specific date over the reported number of COVID-19 cases on the date of death. A limitation of this crude reporting is the misspecification of the population at risk resulting in a more prevalent measure rather than an incident measure of risk [15–18]. We estimated an adjusted CFR (aCFR) by first attributing deaths to the date they were reported positive and then calculating the percentage of COVID-19 mortalities over the reported number of COVID-19 cases on the date the death was reported as a case. The aCFR was calculated as the number of deaths (by date of death tested positive) divided by the number of reported cases on the date the deceased cases tested positive. The infection fatality rate (IFR) is the percentage of COVID-19 mortalities over the true number of COVID-19 cases. The CFR, aCFR and IFR are given by:

$$CFR\;(\%)=\frac{No.\;of\;reported\;COVID-19\;deaths\;(date\;of\;reporting)}{No.\;of\;reported\;COVID-19\;cases\;(date\;of\;reporting)}\;x\;100$$

$$Adjusted\;CFR(\%)=\frac{No.\;of\;reported\;COVID-19\;deaths\;(date\;of\;testing\;positive)}{No.\;of\;reported\;COVID-19\;cases\;(date\;of\;reporting)}\;x\;100$$

$$IFR=\frac{All\;COVID-19\;deaths}{All\;COVID-19\;cases}\;x\;100$$

We approximated the age-stratified IFR using the lowest-non zero aCFR between 1 October 2020 and 31 October 2021. We utilized this period as few deaths (n = 12) occurred before 1 October 2020, and the risk of death is likely different after 31 October 2021 due to the National COVID-19 Immunization Program. The age-stratified aCFR was estimated over a moving 3-month period to stabilize the approximated IFR as there was a low number of deaths reported in some age strata. The lowest age-stratified non-zero aCFR was compared to reported pooled estimates [19, 20].

The ratio of CFR to IFR will equal 1 when all cases are ascertained ($\frac{CFR}{IFR}=1)$. As the number of underestimated cases increases, the CFR also increases, and as such $\frac{CFR}{IFR}>1$. We corrected the observed number of cases to reflect the true number of cases by calibrating the observed cases against an adjustment factor obtained from the ratio of $\frac{CFR}{IFR}$ which is given by:

Etp=Otp*CFRtpIFRp,whereOtp=observedcasesattimetinagegrouppIFRp=Lowestnon-zeroCFRinagegroupp,Et=expectednumberofcasesinagegroupp

The expected distribution of COVID-19 cases from COVID-19 mortalities assumes: i) the estimated lowest non-zero CFR approximates the unknown IFR, ii) All COVID-19 deaths are reported, iv) Exclusion of brought-in-dead mortalities will account for any excess mortality secondary to poor healthcare accessibility, and v) IFR is constant through time. Hospital protocols during this period likely resulted in high ascertainment of COVID-19 related deaths among all deaths. In addition, all reported deaths outside the hospital were tested for SARS-CoV2 using RT-PCR tests. All adjustment factors below one were replaced with one as it is impossible to estimate fewer cases than reported and were likely due to a small number of observed cases. The expected number of infections was rounded to the nearest thousand to ease interpretation. Reported cases and the estimated infections were tabulated by months. A sensitivity analysis was carried out to quantify the effect of hospital death underreporting on aCFR and case estimation. The Farrington algorithm was utilised to model daily age-specific excess mortality counts between January 2020 and September 2021 in Malaysia using age-specific all-cause mortality data from January 2016 to December 2019 [21–27]. A back-projection model following a Poisson process was carried out to estimate the unobserved age-specific death curve on the day a case was reported positive using an empirically estimated time-lagged age-specific delay distribution from reporting to death [28–30]. Excess mortality counts were then utilised in estimating the aCFR and subsequent degree of underestimation. All analysis was carried out using the “tidyverse”, “zoo”, “epitools”, “prevalence”, “boot”, “amelia” and “surveillance” packages in R 4.3.1.

## Results

**Fig 1:**
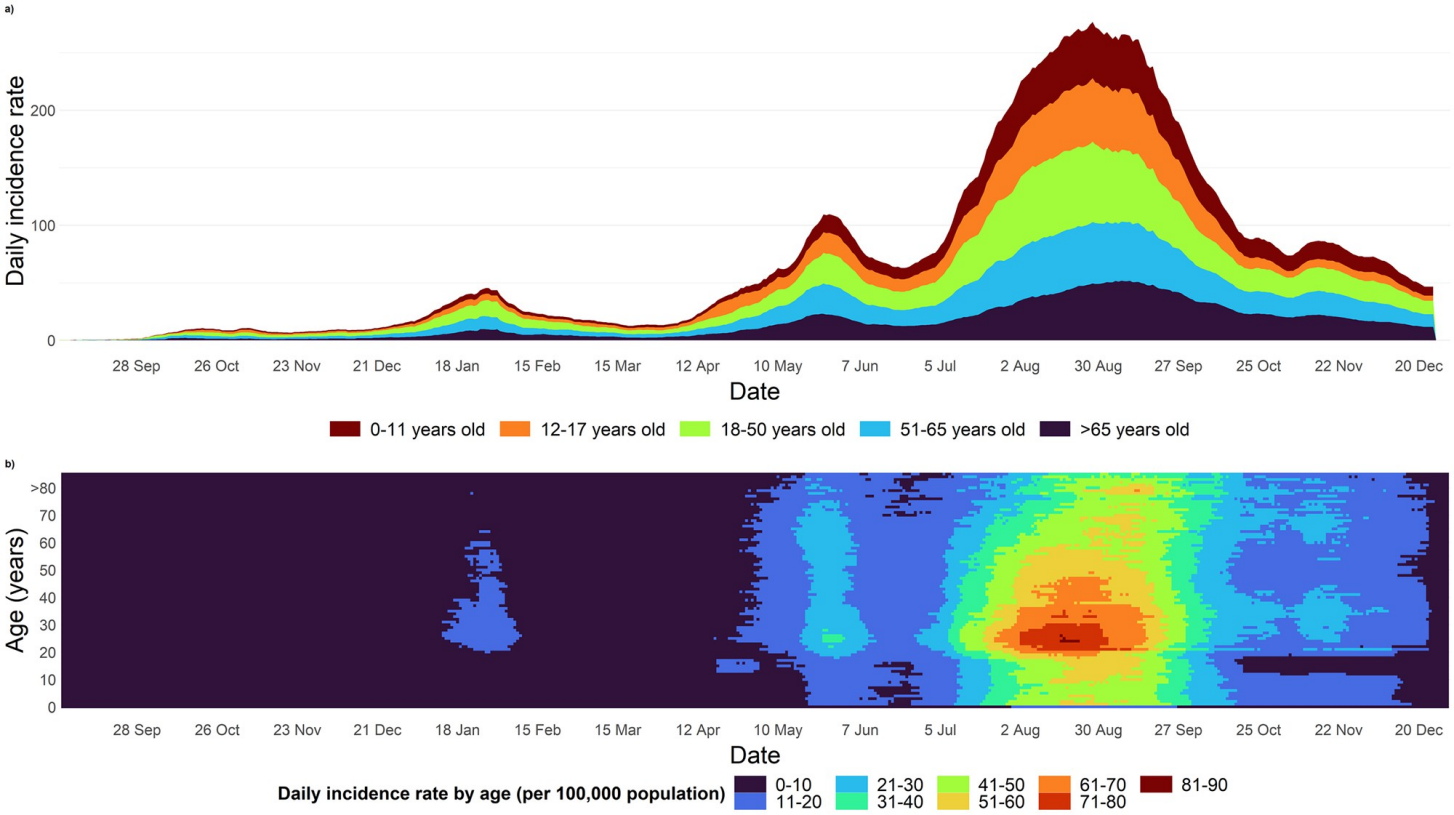
Cases in Malaysia between 1 October 2020–30 December 2021 in Malaysia. a) Daily incidence rate by age categories across time, b) Heatmap of daily incidence rate by age categories across time (Data before 1 September 2020 were removed as deaths were relatively lower to increase visualization quality)

A total of 2.31 million cases were reported in Malaysians between 1 March 2020 and 30 December 2021. Incidence trends were quadrimodal with peaks on 26 March 2020 (n = 235), 30 January 2021 (n = 5,728), 29 May 2021 (n = 9,020) and 12 August 2021 (n = 21,668). Cases aged 18 and 50 had the highest daily reported case density between August and September 2021 (Fig 1).

**Fig 2:**
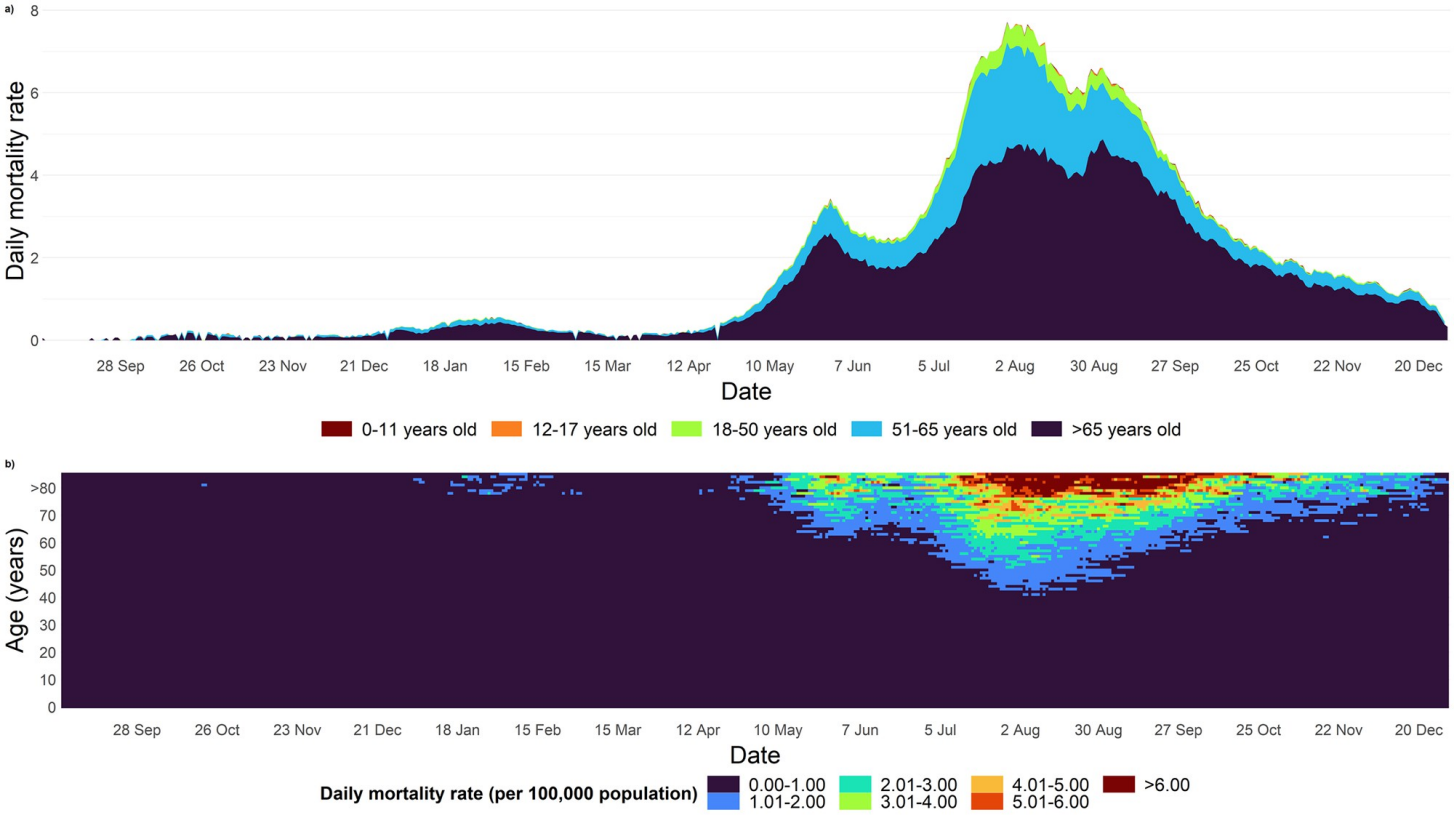
Deaths in Malaysia between 1^st^ October 2020-30^th^ December 2021 in Malaysia. a) Daily deaths by age categories across time, b) Heatmap of daily mortality rate by age categories across time (Data before 1 September 2020 were removed as deaths were relatively lower to increase visualization quality).

A total of 27,711 deaths were reported between 1 March 2020 and 30 December 2021 (Table 1). Mortality trends were quadrimodal with peaks reported on 29 March 2020 (n = 7), 18 February 2021 (n = 25), 2 June 2021 (n = 126), and 9 August 2021 (n = 360). The age-specific mortality rate is highest among individuals aged above 60 between August and September 2021 (Fig 2). The distribution of cases by age remained similar across time.

**Table 1:** Monthly COVID-19 reported deaths between March 2020 and December 2021 in Malaysia

	Total	Age groups (%)				
		0–11 years old	12–17 years old	18–50 years old	51–65 years old	>65 years old
**Total**	27,711	55	42	5,652	8,932	13,030
**Mar-20**	47	0 (0)	0 (0)	14 (29.8)	10 (21.3)	23 (48.9)
**Apr-20**	52	0 (0)	0 (0)	3 (5.8)	20 (38.5)	29 (55.8)
**May-20**	12	0 (0)	0 (0)	3 (25)	5 (41.7)	4 (33.3)
**Jun-20**	5	0 (0)	0 (0)	0 (0)	2 (40)	3 (60)
**Jul-20**	3	0 (0)	0 (0)	0 (0)	1 (33.3)	2 (66.7)
**Aug-20**	3	0 (0)	0 (0)	1 (33.3)	1 (33.3)	1 (33.3)
**Sep-20**	8	0 (0)	0 (0)	1 (12.5)	1 (12.5)	6 (75)
**Oct-20**	102	1 (1)	0 (0)	13 (12.7)	36 (35.3)	52 (51)
**Nov-20**	83	2 (2.4)	1 (1.2)	9 (10.8)	34 (41)	37 (44.6)
**Dec-20**	121	0 (0)	0 (0)	17 (14)	47 (38.8)	57 (47.1)
**Jan-21**	357	0 (0)	0 (0)	52 (14.6)	119 (33.3)	186 (52.1)
**Feb-21**	288	2 (0.7)	0 (0)	29 (10.1)	84 (29.2)	173 (60.1)
**Mar-21**	124	0 (0)	0 (0)	12 (9.7)	33 (26.6)	79 (63.7)
**Apr-21**	243	1 (0.4)	0 (0)	39 (16)	61 (25.1)	142 (58.4)
**May-21**	1,574	1 (0.1)	3 (0.2)	158 (10)	490 (31.1)	922 (58.6)
**Jun-21**	2,313	1 (0)	1 (0)	335 (14.5)	790 (34.2)	1,186 (51.3)
**Jul-21**	5,697	6 (0.1)	6 (0.1)	1,321 (23.2)	2,235 (39.2)	2,129 (37.4)
**Aug-21**	7,368	12 (0.2)	13 (0.2)	2,074 (28.1)	2,468 (33.5)	2,801 (38)
**Sep-21**	4,860	10 (0.2)	10 (0.2)	1,054 (21.7)	1,322 (27.2)	2,464 (50.7)
**Oct-21**	2,217	9 (0.4)	7 (0.3)	271 (12.2)	574 (25.9)	1,356 (61.2)
**Nov-21**	1,347	4 (0.3)	1 (0.1)	144 (10.7)	366 (27.2)	832 (61.8)
**Dec-21**	887	6 (0.7)	0 (0)	102 (11.5)	233 (26.3)	546 (61.6)

**Fig 3:**
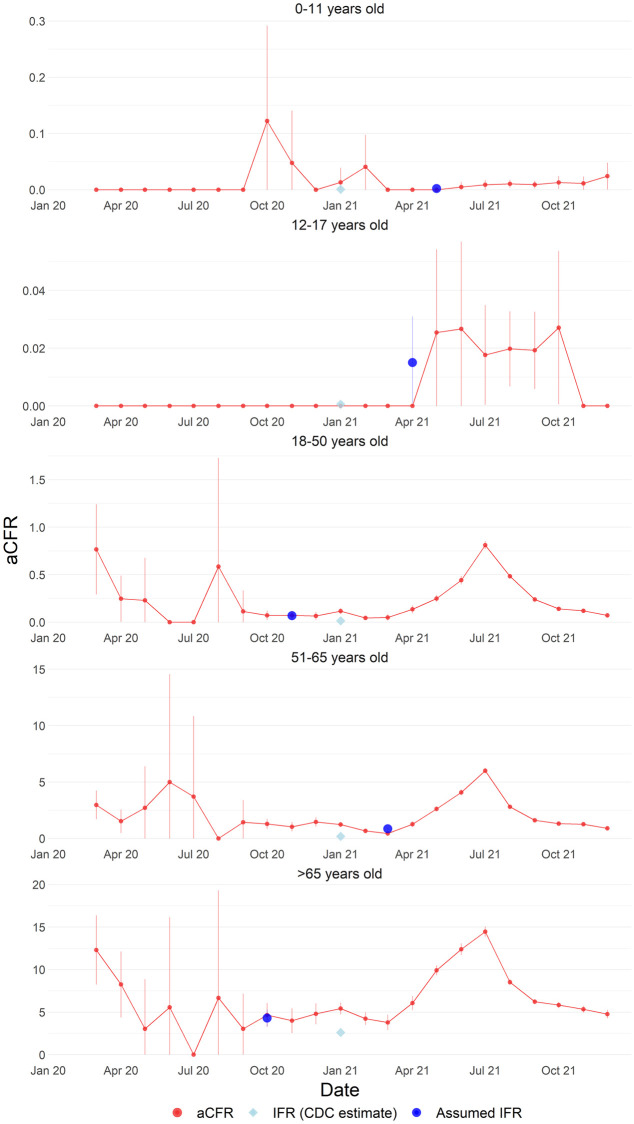
Comparison of reported aCFR of COVID-19 in Malaysia by age categories from 1 March 2020–30 December 2021 in Malaysia compared to assumed IFR and literature-derived IFR

A CFR of 1.0 (95% CI: 0.9–1.0) was reported for Malaysia cumulatively across the study period. Cumulative CFR across the study period is 0.01, 0.02, 0.34, 2.47, and 7.79 in individuals aged between 0–11, 12–17, 18–50, 51–65, and more than 65 years, respectively. The lowest non-zero age specific aCFR utilised for the assumed IFR were 0.002, 0.02, 0.07, 0.85, and 4.3 79 in individuals aged between 0–11, 12–17, 18–50, 51–65, and more than 65 years, respectively. This assumed IFR approximated the pooled age-specific IFR from the literature. The aCFR trends are unimodal in individuals aged 0–11 (peak in October 2020) and 12–17 years old (peak in July 2021). The aCFR trends are bimodal in individuals aged 51–65 and above 65, respectively, with peaks in July 2020 and 2021. The aCFR trends are trimodal in individuals aged 18–50, with peaks in January 2020, July 2020, and July 2021. The aCFR is consistently highest in individuals above 65 years old (Fig 3).

A total of 9,955,000-cases (95% CI: 6,626,000–18,985,000) cases were estimated to have occurred in Malaysia, corresponding to a prevalence of 32.8%. The relative percentage of cases in 0–11, 12–17, 18–50, 51–65, and more than 65 years age groups were 19.9% (n = 1,982,000, 95% CI: 687,000–3,393,200), 2.4% (n = 236,000, 95% CI: 177,000–3,130,000), 66.1% (n = 6,577,000, 95% CI: 4,766,000–10,525,000), 9.1% (n = 901,000, 95% CI: 783,000–1,063,000), and 2.6% (n = 256,000, 95% CI: 210,000–332,000), respectively. Prevalence of infections was highest in those aged 18–50 years (43.9%), followed by those aged 0–11 years (31.9%), 51–65 years (21.9%), above 65 years (12.6%), and 12–17 years (7.9%) (Table 2).

**Table 2:** Monthly reported COVID-19 cases and estimated COVID-19 cases between March 2020 and December 2021 in Malaysia

	Total		Age categories									
			0–11 years old		12–17 years old		18–50 years old		51–65 years old		>65 years old	
	Reported cases	Estimated cases (95% CI)	Reported cases	Estimated cases (95% CI)	Reported cases	Estimated cases (95% CI)	Reported cases	Estimated cases (95% CI)	Reported cases	Estimated cases (95% CI)	Reported cases	Estimated cases (95% CI)
**Total**	2,308.1	9,955 (6,626, 18,985)	360.5	1,982 (687, 3,932)	177.8	237 (178, 3,130)	1319.6	6,577 (4,767, 10,526)	308.6	901 (784, 1,064)	141.6	257 (211, 333)
**March- September 2020**	7.9	31 (24, 47)	0.5	0 (0, 0)	0.5	1 (1, 1)	4.7	25 (18, 40)	1.5	4 (4, 5)	0.6	1 (1, 2)
**October-December 2020**	58.3	210 (107, 385)	6.2	152 (52, 302)	3.8	4 (4, 4)	37.5	38 (38, 60)	8	12 (11, 14)	2.7	3 (3, 4)
**January-March 2021**	152.8	327 (203, 533)	16.6	154 (54, 304)	9	9 (9, 9)	96.8	128 (107, 179)	21.6	26 (24, 29)	8.8	10 (9, 12)
**April-June 2021**	351.8	1267 (932, 2,474)	46.8	75 (47, 125)	29.5	46 (30, 606)	200	904 (650, 1,451)	52.5	186 (161, 219)	23	55 (45, 71)
**July-September 2021**	1,270.1	6906 (4,686, 13,065)	208.2	1,000 (333, 2,000)	109.7	140 (110, 2,100)	725.5	5,048 (3,628, 8,100)	157.5	578 (500, 683)	69.3	141 (114, 182)
**October-December 2021**	467.3	1213 (675, 2,480)	82.2	600 (200, 1,200)	25.3	37 (25, 410)	255.1	433 (327, 695)	67.5	96 (84, 113)	37.2	47 (39, 61)

Notes: All figures are in thousands (,000’s)

**Fig 4:**
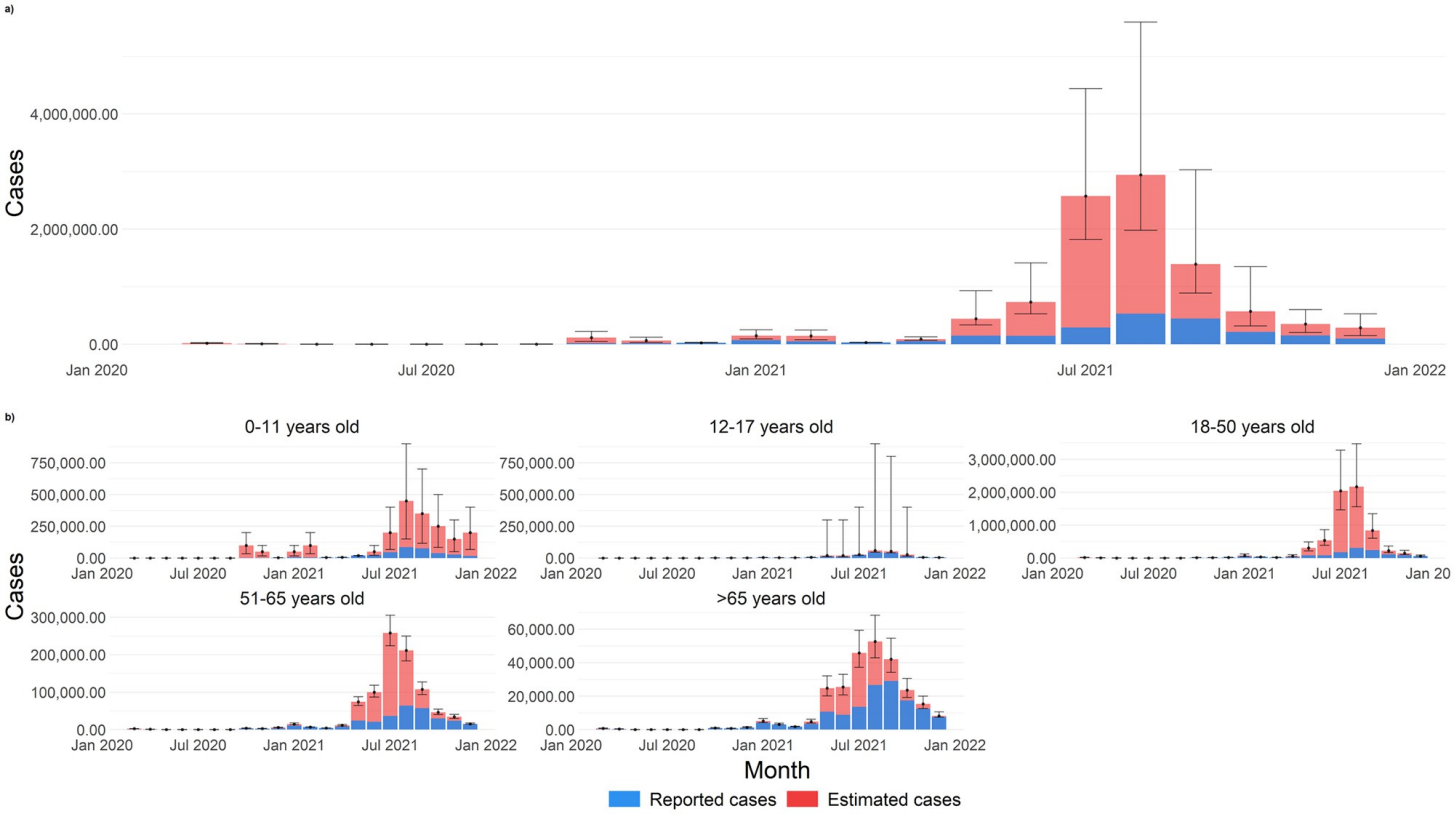
Comparison of reported COVID-19 cases to estimated COVID-19 infections. a) cumulatively and b) by age categories from 1 March 2020–30 December 2021 in Malaysia.

Overall time trends of the age-specific monthly incidence rate are similar to reported infections with four major peaks. Underestimation is highest between June and September 2021. Underestimation of infections was highest in those aged 0–11 years (Adjustment Factor, AF = 5.5; 95%CI: 1.9–10.9), followed by those aged 18–50 years (AF = 5.0; 95% CI: 3.6–7.9), 51–65 years (AF = 2.9; 95% CI: 2.5–3.5), above 65 years (AF = 1.8; 95% CI: 1.9–10.9), and 12–17 years (AF = 1.3, 95% CI:1.0–17.6) The largest number of estimated cases in individuals aged in 0–11, 12–17, 18–50, 51–65, and more than 65 years is observed in August 2021 (n = 450,000, 95% CI: 150,000–900,000), August 2021 (n = 60,000, 95% CI: 45,000–900,000), August 2021 (n = 2,166,000, 95% CI: 1,557,000–3,476,000), July 2021 (n = 258,000, 95% CI: 223,000–305,000) and August 2021 (n = 52,000, 95% CI: 42,871–68,366), respectively (Fig 4)

Population prevalence between 1 January 2020 and 30 September 2021 is estimated to be 25.8% (95% CI: 19.6–54.4) corresponding to 7,835,000-cases (95% CI: 6,665,000–10,994,000), when excess-counts based IFRs are utilised. This compared to a population prevalence of 28.8% (95% CI: 22.0–36.3) corresponding to 8,741,000-cases (95% CI: 5,952,000–16,504,000) when reported-deaths based IFRs were utilised over the same period (Table 3).

**Table 3:** Comparison of reported COVID-19 cases with estimated COVID-19 cases based on reported mortality and excess counts between March 2020 and October 2021 in Malaysia

	Observed Prevalence, %	Estimated Prevalence Model 1, % (95% CI)	Estimated Prevalence Model 2, % (95% CI)
**Age categories, years**			
**Overall**	7.6	28.8 (19.6–54.4)	25.8 (22.-36.3)
**0–11**	5.8	22.2 (7.8–43.9)	21.7 (15.8–36.9)
**12–17**	6.0	6.7 (5.2–91.3)	42.6 (27.3–102.3)
**18–50**	8.8	41. (29.6–65.6)	25.9 (24.5–27.6)
**51–65**	7.5	19.6 (17.-23.1)	18.8 (17.-21.2)
**>65**	6.9	10.3 (8.4–13.3)	27.7 (24.6–31.6)

Notes: Model 1 utilises reported-deaths based approximations of the IFR Model 2 utilises excess-mortality counts based approximations of the IFR

All figures are in thousands (,000’s)

## Discussion

An estimated 32.8% (9.95 million) of the population are likely to have been infected, and 23.2% of COVID-19 infections were reported between March 2020-December 2021. The adjustment factor for the burden of illness varied by time and age group. These results suggest that community immunity is higher than expected.

The number of infections is estimated to be, on average, 4.3 times (Range = 1–8.8) the number of reported cases in Malaysians, with variations by period and age group. The overall underestimation in Malaysia is comparable to estimations in the United States (US) across time and age strata [31, 32]. Underestimation was estimated to be nine times (90% CI: 4–14) higher than reported cases in another IFR-based adjustment in the US [9]. Another global modelling study reported that the true number of infections was 1.4 to 18 times higher than reported cases with heterogeneity between countries [15]. The degree of underestimation is comparable in many of these settings to the findings observed in our study [6,7,33].

Over the study period, an estimated 32.8% of the population is approximated to have been infected. Estimates of seroprevalence over smaller geographical localities and periods are consistent with estimates here [15,34–36]. However, comparisons of the national-level period prevalence estimates were not carried out due to the sparse availability of published literature.

Age-specific prevalences between Malaysia and the United States (US) are comparable except for those above 65 years [31,32]. This may be due to lower aCFR estimations when reported deaths are utilised, as an increase in the number of estimated cases in individuals aged above 65 is observed when excess mortality counts were utilised. Nonetheless, per capita differences remain even when excess mortality counts were utilised. This residual difference of higher prevalence of infection among those above 65 years in the US may be due to the higher proportion of elderly under nursing or institutional care. Malaysia also reports high prevalences in the youngest age groups, with a significant degree of these infections being underestimated [37]. Explanations for this phenomenon include the possibility of preferential testing of older age groups due to their higher risk of severe disease [38]. Changes in this age dynamic have also been reported in countries that have changed testing strategies [39]. Lower susceptibility in younger age groups and a higher propensity to be asymptomatic have also been proposed as an explanation for this dynamic [40]. One final possibility is the use of long-term school closures within many settings, particularly in lower-income countries, which has been suggested to be very effective in reducing transmission [41]. The lack of human-human interaction between younger individuals over a prolonged period, such as has been observed in Malaysia, could potentially amplify this age dynamic.

Published estimates of the true burden of disease have utilized diverse methodologies, and these published findings have primarily been within the global north. These include: i) the use of random population sampling [6,42], ii) seroprevalence studies [43,44], iii) ILI surveillance-based models to estimate prevalence [9,45], iv) crowdsourced data [46], v) testing data based adjustments using probabilistic bias analysis [33] or post-sampling stratification and reweighting [47], and vi) mortality-data based methods such as mechanistic disease models [15], vii) statistical curve fitting [48], viii) mortality mapping using Bayesian frameworks [9], and ix) combination methods; combining mechanistic models, random sampling, and other data sources with the IFR [7]. The methodology proposed here is advantageous to the global South, where estimates of the true burden of disease and resources remain scarce.

There are several limitations to this analysis. Firstly, the CFR may deviate away from the unknown IFR by changes in the testing strategy, unreliable vital statistics data, saturation of surveillance systems, the fluidity of disease transmission, virulence, virus genotype, inability to provide adequate care, availability of resources including human capital, heterogeneities in the distribution of medical comorbidities, and changes in immunity due to vaccination [49]. We utilized an approximated IFR estimated from the lowest non-zero, age-specific aCFR as the CFR has been shown to approximate the IFR when the CFR is smaller [50]. These approximated IFR estimates were similar to published pooled IFR estimates [51–54].

Second, we did not quantify the individual effect of various factors that drive the underestimation of COVID-19 incidence in Malaysia. Selection biases, including issues of access, may lead to varying levels of testing over time and space. Testing strategies may also modify the detection of disease within a population. Asymptomatic and pre-symptomatic transmission, variations in transmissibility, and long-tailed incubation periods further complicate the ascertainment of disease [51–54]. Misclassification biases are driven by the accuracy of tests, with studies suggesting nucleic acid amplification (NAA) test sensitivity ranging from 63% to 89% and NAA test specificity of almost 99% [6,9]. These biases limit a surveillance system’s ability to ascertain the true burden of COVID-19 infections [45].

Third, the aCFR was estimated using reported deaths instead of the number of excess deaths. The estimated infection may be underestimated using the aCFR compared to excess deaths in countries with poor COVID-19 specific mortality reporting. However, the Malaysian National Death register has a high ascertainment coverage [55,56], and sensitivity analysis revealed small differences in the prevalence estimate using excess counts (25.8%) and reported deaths (28.8%). Fourth, the incidence density estimates utilised the mid-year population as the population at risk. However, this assumes that infections and vaccinations do not provide complete immunity to the SARS-CoV2 virus. Violation of this assumption may lead to underestimation of the Incidence density. Finally, we also used a multiplier model approach instead of a mechanistic approach, limiting its utility in forecasting future disease dynamics.

## Conclusion

The characterization of the true burden of disease is essential in developing and implementing policy measures and allocating resources. These estimations highlight a more accurate infection burden in Malaysia. It provides the first national-level estimates of prevalence in Malaysia that are adjusted for underdiagnosis. The higher underestimation of infections during April-September 2021 coincided with sustained higher community transmission and higher healthcare utilization.

Naturally acquired community immunity is still low but is likely to increase secondary to an Omicron-fuelled infection surge. Booster doses may further hasten an equilibrium between the SARS-CoV-2 virus and its human host. Such an equilibrium should mark the start of an endemic state. Future variants may upend this equilibrium and necessitate periodic mitigation of disease transmission. Population estimates of the infection burden are critical to determine the need for booster doses and public health measures.

## Supporting information

S1 Appendix Adjudication of deaths in Malaysia. (DOCX)
