# Supplementary material for: Estimating the infection burden of COVID-19 in Malaysia
Source: PLoS Negl Trop Dis. 2022 Nov 8;16(11):e0010887. doi: 10.1371/journal.pntd.0010887 (PMC9642899; doi:10.1371/journal.pntd.0010887)
Supplement: S1 Appendix — (DOCX) [file pntd.0010887.s001.docx]

**Estimating the infection burden of COVID-19 in Malaysia**

Vivek Jason Jayaraj^1,2^, Chiu-Wan Ng ^1^, Awang Bulgiba^1^, Maheshwara Rao Appannan^2^, Sanjay Rampal^1*^

^1^Centre for Epidemiology and Evidence-based Practice, Department of Social and Preventive

Medicine, Faculty of Medicine, University of Malaya, 50603 Kuala Lumpur, Malaysia

^2^Ministry of Health Malaysia, Putrajaya, Malaysia

*Corresponding author

Email: srampal@ummc.edu.my

**Appendix**

Appendix 1: Adjudication of deaths in Malaysia

1. **Definition of Term**
2. Inpatient
   - - Refers to a patient who was brought into hospital without any recordable vital signs suggesting life as verified by the attending physician
3. Brought-in-dead (BID)
   - - Refers to a patient who stays in hospital while under treatment or reaches the hospital alive

## Classification of "Death Due to COVID-19"

1. Criteria for Inpatient:

- COVID-19 positive case that has been confirmed through laboratory tests (RTPCR/ RTK/ confirmatory test, which has been recognized by the State);
- Death resulting from clinically compatible disease;
- No clear alternative cause of death that is not related to COVID-19 infection (examples: death due to trauma, road traffic accident, suicide, etc.);
- No period of complete recovery from COVID-19 between illness and death;
- Even with pre-existing disease (e.g., cancer), it is death due to COVID-19 if the reason for the severe course is due to COVID-19 infection; and
- Death of a positive case within 30 days from a positive result.

## Criteria for Brought-in-dead (BID):

- - - If a post-mortem is conducted, the classification of COVID-19 death shall be in accordance with the autopsy death report (Gold Standard).
    - If the autopsy is not performed, death due to COVID-19 must meet the following criteria:
      - COVID-19 positive case that has been confirmed through laboratory test (RTPCR/ Gene Expert/ confirmatory test which has been recognized by the State) with CT value (if the CT value is available);
      - Has an epidemiological chain (according to the Epidemiology Link criteria for COVID-19 cases);
      - Supportive Clinical History (if available);
      - Supportive radiological investigation (where applicable) (examples: chest x-ray, post-mortem CT scan, etc.), which shows signs of COVID- 19 infection (reported by qualified personnel), can be used depending on the local and current situations; and
      - Death of a positive case within 30 days from a positive result.

1. Classification of "Death with COVID-19"
   COVID-19 related death that does NOT meet the criteria in paragraph 4 would be
   Classified as "Death with COVID-19" instead of "Death Due to COVID-19". These
   Deaths are NOT included in the National COVID-19 Mortality Statistics.
